# Progress in Phototherapy and Electro-Therapeutics

**Published:** 1903-09-26

**Authors:** 


### Progress in Phototherapy and Electro-Therapeutics.

**Radium.**—The greatest interest in the progress in radiotherapy is focussed upon radium and its salts. While chemists are telling us of the possibilities in their use, the profession is not backward in experimenting with this new therapeutic agent. A new possibility is introduced by Frederick Soddy [1] who discusses the possibility of using the radio-active emanations from thorium and radium in the treatment of phthisis. These emanations are a separate phenomenon from the rays, being of a gaseous character, and, although in infinitesimal quantities, are capable of giving out on their own account similar rays to thorium and radium themselves. The radio-activity is very great and unceasing, but the gases under certain physical conditions and in certain compounds are "occluded." To liberate them freely the best and most certain method is to make a solution of the salt in water. In the dry state the emanations rarely escape, but directly the salt is dropped into water the stored up, occluded, gas instantly escapes into the superjacent air.

Thorium and radium resemble one another nearly, they are both spontaneously radio-active, though radium is about a million times more powerful than thorium. These elements both produce radio-active emanations. When a current of air is passed into a solution of radium salt, and is stored in a gas-holder, the storage is not permanent, one-half of the emanation disappearing every four days, so that in eight days only one-quarter of the original is left, and in 12 days only one-eighth, etc. In the meantime the original salt in solution has developed a fresh store, the quantity produced equalising the quantity lost from the store. With thorium the phenomenon is the same, only instead of half the emanation being lost in four days it is lost in a minute. Radium takes three weeks and thorium only a few minutes to regain all its emanations. By considering this the dosage can be regulated. Consumptive patients should be made to breathe continuously through a thorium solution to secure the most efficient action, and the longer the inhalation the greater the action. In the case of radium, however, after the patient has breathed in all the emanation from any quantity of radium, further inhalation is without effect till more emanation has been stored. If the patient breathes in once a day and then leaves the bottle stoppered tightly, he will get the maximum that this amount of radium can supply. These emanations wherever they come in contact with matter leave behind upon it a fine film of radio-active matter, which disappears in a definite ratio, though gradually. The excited activity of thoriun falls to one half its initial intensity in eleven hours; that of radium emanation in three to four hours. This takes place without regard to environment or the action of any known force which may be brought to bear upon the radio-active processes, and regardless of what chemical compound of the elements is used. Though a milligram of radium is roughly equivalent to a kilogram of thorium in radio-activity, the cost of a kilogram of thorium nitrate (about £2) is only two or three times as great as the cost of a milligram of radium bromide. Thorium can be obtained in quantity, radium is very hard to obtain. Though as far as surface is concerned the effect is proportionate to the area, with regard to the emanation it is dependent upon the amount used. Hence thorium though feebler, is more continuous in its action, and being within the reach of everyone, can be tried first and increased indefinitely as required. If this can be tolerated satisfactorily, the very powerful agent radium can be used with caution. As to whether this will be of use therapeutically has yet to be ascertained by experiment. It seems that considerable time is required for the radium rays to kill the germs experimented on by Pfeiffer and Friedberger.[2] They exposed typhoid and cholera cultures to the action of the rays generated by 25 milligrams of radium bromide in a vulcanite capsule with a mica plate, the whole being protected by a brass cover with a central opening. The rays generated were capable of penetrating 5·6 cms. of bronze plate. No result was obtained at 6-10 cms. on cultures of typhoid and cholera. In 48 hours a central circle 2 cms. in diameter clear of bacilli was found in a plate culture of B. Typhosus, when the rays were acting at 1 cm. distance, the plate outside the circle being thick with bacillary growth. That the gelatine itself was not changed was shown by a later inoculation of the clear space successfully starting a culture. The same happened with cholera bacilli. Anthrax spores were killed in three days, but two days were not sufficient. The authors think that some use might be made of this therapeutically, due cognisance being taken of the possible destruction of tissue cells. That this destruction can be severe was proved by Macintyre [3], who placed two or three milligrams against the skin of his arm, so arranged that the rays had to traverse a layer of air and one of mica, for an hour. The immediate result was erythema; next day a dermatitis ensued on an area six times as great as that of the irradiation. On the third day the epidermis was found to be destroyed, and on the fourth a small ulcer had developed in the central patch, and there was for days afterwards slight though distinct pain. In his later report Macintyre [4] states that healing had not taken place in two months, and that some time yet would be required. In Becquerel's case burning of the skin occurred though the rays passed through a glass tube kept in the pocket. Macintyre finds three groups of rays in the radiations from radium—($a$) infinitely small positively-charged atoms flying at a great speed which can be measured, and the result seen by the bombardment of zinc sulphide screens; ($\beta$) rays which seemingly correspond to the cathodal rays of a Crookes's tube, also described by Leonard as passing through an aluminium window in the same; ($\gamma$) rays which evidently correspond to the $x$-rays. The $\alpha$-rays are easily stopped by thin interposed substances. The $\beta$-rays have great penetrating power. Radium and its salts have great power of emitting heat. If enclosed in a heat insulating apparatus 5° F. can be registered above the surrounding things.

Radium also has a power inimical to life, as has been proved by E. S. London,[5] who tested the effect on mice of prolonged irradiation from 30 milligrams of radium bromide. He inverted the box containing the radium which was provided with a mica top, on the wire roof of the cage and watched the effect. Symptoms of reddening of the ears and blinking of the eyes appeared on the third day; sleepiness followed with refusal of food, general slackness of movements, coma, associated with paralysis of the extremities, chiefly the hind limbs. The reflexes were often increased and the respiration slowed. Functions of the brain and spinal cord ceased and death followed on paralysis of respiration on the fourth or fifth day. The chief lesions were found in the skin and subcutaneous tissue, and in the cortex of the brain. Arterial blood is darkened by the rays.

London has also discovered that persons with only a faint perception of light can receive light impressions when radium is brought near to the eye, and this even in bright light. Persons totally blind cannot see it at all. Persons with perception of light but none of form can detect the shape of things either behind or in front of the screen. He thinks that there is here a possibility of teaching such blind persons to write or draw. The position of radium can be detected with covered eyes and even when the box is held behind the head.

Macintyre,[6] using an apparatus to keep the salt at a distance from the tissues, has tried the effect of radium upon diseased areas. His applications were from 20 to 40 minutes. In a case of lupus of the hand daily exposures were made of from 20 to 30 minutes, according to the reaction. He obtained evidence of healing in a week, and in three weeks the lesion ($\frac{1}{2}$ inch in diameter), had disappeared. Lupus of the left ala of the nose in a woman, æt. 28, area 1 inch, had practically disappeared in four weeks. This was a recurrence after Finsen treatment a year previously. In a case of rodent ulcer the improvement was obvious in a fortnight. We are not yet in possession of sufficient data for the satisfactory use of radium, nor do we know what nature of cicatrix is left behind.

Hallopeau and Gadaud[7] have treated a patient with verrucose lupus of the hand with radium, and much improvement followed. The first application was made for 72 hours and produced a lasting discoloration and ulceration ensued 15 days later. Two months later another application of radium was made for 120 hours and this produced a permanent loss of substance. Seven other applications for 24 hours at a time left no trace. The improvement was very considerable, the verrucose elevations had disappeared leaving a smooth cicatrix of healthy aspect. Danlos reports four cases treated in the same manner and holds that there is a great future for radium in dermatotherapy. Its employment is justifiable in erythematous lupus, lupus vulgaris, hypertrichosis, superficial cancer, nævi, etc.

[1] Brit. Med. Jour., July 25. [2] Ibid., Aug. 22. [3] Ibid., June 6. [4] Ibid., July 25. [5] Ibid. [6] Ibid. [7] Amer. Jour. Med. Sci., Jan.

*(To be continued.)*

